# Diversity and Composition of Airborne Fungal Community Associated with Particulate Matters in Beijing during Haze and Non-haze Days

**DOI:** 10.3389/fmicb.2016.00487

**Published:** 2016-04-14

**Authors:** Dong Yan, Tao Zhang, Jing Su, Li-Li Zhao, Hao Wang, Xiao-Mei Fang, Yu-Qin Zhang, Hong-Yu Liu, Li-Yan Yu

**Affiliations:** China Pharmaceutical Culture Collection, Institute of Medicinal Biotechnology, Chinese Academy of Medical Sciences & Peking Union Medical CollegeBeijing, China

**Keywords:** airborne fungi, fungal community composition, PM2.5, PM10, TSP, haze, high-throughput sequencing

## Abstract

To assess the diversity and composition of airborne fungi associated with particulate matters (PMs) in Beijing, China, a total of 81 PM samples were collected, which were derived from PM2.5, PM10 fractions, and total suspended particles during haze and non-haze days. The airborne fungal community in these samples was analyzed using the Illumina Miseq platform with fungi-specific primers targeting the internal transcribed spacer 1 region of the large subunit rRNA gene. A total of 797,040 reads belonging to 1633 operational taxonomic units were observed. Of these, 1102 belonged to Ascomycota, 502 to Basidiomycota, 24 to Zygomycota, and 5 to Chytridiomycota. The dominant orders were Pleosporales (29.39%), Capnodiales (27.96%), Eurotiales (10.64%), and Hypocreales (9.01%). The dominant genera were *Cladosporium, Alternaria, Fusarium, Penicillium, Sporisorium*, and *Aspergilus*. Analysis of similarities revealed that both particulate matter sizes (*R* = 0.175, *p* = 0.001) and air quality levels (*R* = 0.076, *p* = 0.006) significantly affected the airborne fungal community composition. The relative abundance of many fungal genera was found to significantly differ among various PM types and air quality levels. *Alternaria* and *Epicoccum* were more abundant in total suspended particles samples, *Aspergillus* in heavy-haze days and PM2.5 samples, and *Malassezia* in PM2.5 samples and heavy-haze days. Canonical correspondence analysis and permutation tests showed that temperature (*p* < 0.01), NO_2_ (*p* < 0.01), PM10 (*p* < 0.01), SO_2_(*p* < 0.01), CO (*p* < 0.01), and relative humidity (*p* < 0.05) were significant factors that determine airborne fungal community composition. The results suggest that diverse airborne fungal communities are associated with particulate matters and may provide reliable data for studying the responses of human body to the increasing level of air pollution in Beijing.

## Introduction

In recent years, particulate matters (PMs) have become a major air pollutant in densely populated cities or urban areas, and their impact to the public health may be profound (Zhang et al., 2010; Cheng et al., 2013; Evans et al., 2013; Beelen et al., 2014; DeFranco et al., 2015). PM pollutants are usually highly toxic to humans and can cause severe sickness or even death. Different PMs may affect various parts of the human body. For example, total suspended particles (TSP, <100 μm) cause skin allergy, PM10 (<10 μm) deposit mainly in the head airways, and PM2.5 (<2.5 μm) are more likely to penetrate and deposit deeper in the tracheobronchial and alveolar regions (Brook et al., 2004). Historical data suggest that exposure to high level of atmospheric PM pollutants, particularly PM2.5, increases morbidity and mortality, and thereby shortening the life span (Hunt et al., 2003; Pope et al., 2009).

While the physical and chemical properties of PM pollutants have been extensively studied (Putaud et al., 2004; Kim et al., 2005; Kuhn et al., 2005; Bressi et al., 2013; Li et al., 2013; Huang et al., 2014; Yang et al., 2016), relatively little is known about microbes associated with PMs, especially airborne fungi. Fungal spores can account for large proportions of air PMs, and some fungi are major pathogens or allergens for humans, animals, and plants. Moreover, air is the primary medium for fungal dispersal (Adhikari et al., 2004). Hitherto, a few studies carried out provide only limited insight into airborne fungi associated with PMs. Airborne fungi associated with PMs have been reported from different regions of the world, including Beijing (China; Cao et al., 2014; Gao et al., 2015), Xi'an (China; Li et al., 2015), Hong Kong (China; Woo et al., 2013), Denver and Greeley (USA; Bowers et al., 2013), New Haven (USA; Yamamoto et al., 2012), Berkeley (USA; Adams et al., 2013), Rehovot (Israel; Dannemiller et al., 2014), Graz (Austria; Haas et al., 2013), Manaus (Brazil; Womack et al., 2015), and Mainz (Germany; Fröhlich-Nowoisky et al., 2009).

Most previous studies focused on the fungal diversity associated with PMs using traditional isolation methods (Awad et al., 2013; Haas et al., 2013; Almaguer et al., 2014; Gao et al., 2015; Li et al., 2015). However, the cultured method cannot reflect the actual diversity of airborne fungi because of its selectivity. In addition, a few studies surveyed PMs-associated airborne fungi using conventional DNA-based molecular methods (e.g., cloning approaches, q-PCR, DGGE; Fröhlich-Nowoisky et al., 2009; Li et al., 2010; Rittenour et al., 2014). In recent years, diversity and community composition of PMs-associated airborne fungi have been revealed by high-throughput sequencing (Yamamoto et al., 2012, 2014; Woo et al., 2013; Cao et al., 2014; Dannemiller et al., 2014; Emerson et al., 2015; Womack et al., 2015), which can significantly enhance the characterization of fungal diversity compared to traditional methods.

Beijing, the capital of China with a population of over 20 million, in recent years, has been suffering from frequent smog events caused mainly by PMs (Zhang et al., 2010, 2012; Cheng et al., 2013; Cao et al., 2014; Gao et al., 2015). While previous findings have improved our understanding of air fungal diversity in urban area of Beijing (Cao et al., 2014; Gao et al., 2015), the fungal community associated with PMs during haze and non-haze days are not well-understood. The aim of this study was to use high-throughput sequencing to investigate the PMs-associated air fungal community in Beijing to address the following questions: (1) what are air fungal diversity and community composition associated with PMs, (2) does the fungal community composition differ among various PMs (PM2.5, PM10, and TSP), and (3) does the fungal community composition vary among different air quality levels?

## Materials and methods

### Sample collection

Samples for PM2.5, PM10, and TSP were collected from the roof top of the Conference Building at Institute of Medicinal Biotechnology, Chinese Academy of Medical Sciences (39°52′43″N, 116°23′21″E, ~8 m above the ground, ~400 m from Temple of Heaven Park), an area without major pollution sources nearby. Sampling was conducted by three portable ambient air samplers (Air Metrics, USA), one of which, impactors were removed from the filter holder for TSP samples, the other one was assembled with PM10 impactor for PM10 samples, and another one was assembled with both PM10 and PM2.5 impactors for PM2.5 samples. Ambient air was drawn at an average flow rate of 5 L/min for 24 h (12:00 p.m. to 12:00 p.m. at next day) per sampling day. PM samples were collected on 47-mm quartz aerosol collection filters (Pall, USA). Prior to sampling, all the filters were sterilized by autoclaving at 121°C for 20 min. The filter holders were cleaned with 75% ethanol and all the tools used for changing new filters were autoclaved every day to avoid contamination. After sampling, the filters were kept in a 2-ml centrifuge tube and stored at −20°C.

A total of 81 PM samples were collected for 27 days (June–November 2014) during different air quality levels (Table 1). The air quality index (AQI), which is an index for reporting daily air quality (Wang et al., 2006), was used to indicate the pollutant level. We define a day with AQI lower than 100 as a non-haze day, that with AQI in the range of 100–200 as a light-haze day, and that with AQI higher than 200 as a heavy-haze day. The environmental variables such as PM2.5, PM10, CO, SO_2_, and NO_2_ were recorded from the monitoring data of Temple of Heaven Park site (~800 m from sampling site) of Beijing Municipal Environmental Monitoring Center (http://zx.bjmemc.com.cn/). Temperature (Temp) and relative humidity (RH) were recorded according to the reports of Chinese National Meteorological Center (http://www.nmc.cn/). The air environmental variables are listed in Table S1.

**Table 1:** **Summary data for sequencing data from the 81 particulate matter samples in the present study**.

**Sample code**	**Sample date**	**Particulate matter type**	**AQI/Day type**	**Number of OTUs**	**Good's coverage estimator (%)**	**Chao1**	**Shannon**
1A	6/2/2014	PM2.5	45/Non-haze	82	99.73	107	1.08
1B	6/2/2014	PM10	45/Non-haze	165	99.44	216	2.25
1C	6/2/2014	TSP	45/Non-haze	244	99.44	274	2.83
2A	6/3/2014	PM2.5	63/Non-haze	93	99.96	96	3.25
2B	6/3/2014	PM10	63/Non-haze	121	99.59	167	1.87
2C	6/3/2014	TSP	63/Non-haze	115	99.60	168	1.74
3A	6/4/2014	PM2.5	83/Non-haze	64	99.98	64	2.85
3B	6/4/2014	PM10	83/Non-haze	104	99.75	122	2.26
3C	6/4/2014	TSP	83/Non-haze	86	99.63	156	1.34
4A	6/5/2014	PM2.5	104/Light-haze	46	99.98	46	2.32
4B	6/5/2014	PM10	104/Light-haze	92	99.93	95	2.56
4C	6/5/2014	TSP	104/Light-haze	113	99.92	115	2.21
5A	6/7/2014	PM2.5	32/Non-haze	92	99.76	110	2.08
5B	6/7/2014	PM10	32/Non-haze	154	99.35	215	2.22
5C	6/7/2014	TSP	32/Non-haze	103	99.57	151	1.85
6A	6/11/2014	PM2.5	46/Non-haze	82	99.96	83	2.94
6B	6/11/2014	PM10	46/Non-haze	218	99.79	226	3.11
6C	6/11/2014	TSP	46/Non-haze	188	99.48	218	2.23
7A	6/12/2014	PM2.5	77/Non-haze	40	100.00	40	2.27
7B	6/12/2014	PM10	77/Non-haze	104	99.91	113	2.29
7C	6/12/2014	TSP	77/Non-haze	137	99.70	151	2.12
8A	6/26/2014	PM2.5	109/Light-haze	106	99.90	112	2.59
8B	6/26/2014	PM10	109/Light-haze	256	99.44	294	2.61
8C	6/26/2014	TSP	109/Light-haze	194	99.48	234	1.85
10A	7/3/2014	PM2.5	269/Heavy-haze	59	99.95	60	2.97
10B	7/3/2014	PM10	269/Heavy-haze	59	99.97	59	3.06
10C	7/3/2014	TSP	269/Heavy-haze	81	99.91	84	2.22
11A	7/4/2014	PM2.5	216/Heavy-haze	54	99.95	60	2.21
11B	7/4/2014	PM10	216/Heavy-haze	81	99.94	84	2.52
11C	7/4/2014	TSP	216/Heavy-haze	117	99.62	142	1.23
12A	7/5/2014	PM2.5	210/Heavy-haze	90	99.96	91	2.81
12B	7/5/2014	PM10	210/Heavy-haze	110	99.92	131	3.2
12C	7/5/2014	TSP	210/Heavy-haze	81	99.93	84	1.83
13A	9/16/2014	PM2.5	60/Non-haze	127	99.93	128	2.86
13B	9/16/2014	PM10	60/Non-haze	184	99.49	224	2.72
13C	9/16/2014	TSP	60/Non-haze	143	99.82	150	1.96
14A	9/18/2014	PM2.5	104/Light-haze	34	99.96	35	0.43
14B	9/18/2014	PM10	104/Light-haze	63	99.91	68	1.7
14C	9/18/2014	TSP	104/Light-haze	89	99.80	106	1.59
16A	9/21/2014	PM2.5	116/Light-haze	101	99.88	107	2.92
16B	9/21/2014	PM10	116/Light-haze	146	99.67	174	2.14
16C	9/21/2014	TSP	116/light-haze	139	99.46	196	1.59
17A	9/27/2014	PM2.5	130/Light-haze	112	99.74	145	2.12
17B	9/27/2014	PM10	130/Light-haze	113	99.54	171	1.65
17C	9/27/2014	TSP	130/Light-haze	215	99.48	249	2.39
18A	9/29/2014	PM2.5	43/Non-haze	175	99.79	183	2.41
18B	9/29/2014	PM10	43/Non-haze	132	99.35	225	1.41
18C	9/29/2014	TSP	43/Non-haze	142	99.31	238	1.55
19A	9/30/2014	PM2.5	74/Non-haze	50	99.94	60	3.06
19B	9/30/2014	PM10	74/Non-haze	177	99.57	201	2.09
19C	9/30/2014	TSP	74/Non-haze	144	99.49	200	1.92
20A	10/8/2014	PM2.5	328/Heavy-haze	133	99.68	162	2.75
20B	10/8/2014	PM10	328/Heavy-haze	119	99.84	125	1.34
20C	10/8/2014	TSP	328/Heavy-haze	130	99.51	186	1.85
21A	10/9/2014	PM2.5	352/Heavy-haze	82	99.93	90	3.2
21B	10/9/2014	PM10	352/Heavy-haze	123	99.93	128	3.94
21C	10/9/2014	TSP	352/Heavy-haze	120	99.84	128	2.4
22A	10/12/2014	PM2.5	15/Non-haze	92	99.57	158	0.94
22B	10/12/2014	PM10	15/Non-haze	219	99.39	268	2.1
22C	10/12/2014	TSP	15/Non-haze	202	99.17	276	2.03
23A	10/17/2014	PM2.5	151/Light-haze	216	99.44	253	2.99
23B	10/17/2014	PM10	151/Light-haze	247	99.04	346	2.19
23C	10/17/2014	TSP	151/Light-haze	155	99.35	230	1.89
24A	10/18/2014	PM2.5	303/Heavy-haze	47	99.93	51	2.74
24B	10/18/2014	PM10	303/Heavy-haze	260	99.40	304	3.29
24C	10/18/2014	TSP	303/Heavy-haze	206	99.26	297	2.3
25A	10/19/2014	PM2.5	211/Heavy-haze	124	99.93	126	3.26
25B	10/19/2014	PM10	211/Heavy-haze	179	99.86	183	3.61
25C	10/19/2014	TSP	211/Heavy-haze	214	99.67	235	2.94
26A	10/20/2014	PM2.5	144/Light-haze	159	99.74	182	2.84
26B	10/20/2014	PM10	144/Light-haze	184	99.46	227	2.11
26C	10/20/2014	TSP	144/Light-haze	80	99.93	84	2.59
28A	10/30/2014	PM2.5	213/Heavy-haze	135	99.89	138	2.88
28B	10/30/2014	PM10	213/Heavy-haze	159	99.91	165	2.75
28C	10/30/2014	TSP	213/Heavy-haze	185	99.74	197	2.84
29A	10/31/2014	PM2.5	175/Light-haze	88	99.95	90	3.15
29B	10/31/2014	PM10	175/Light-haze	161	99.73	186	2.76
29C	10/31/2014	TSP	175/Light-haze	241	99.47	275	2.98
30A	11/1/2014	PM2.5	31/Non-haze	46	99.94	48	0.47
30B	11/1/2014	PM10	31/Non-haze	285	99.51	306	2.69
30C	11/1/2014	TSP	31/Non-haze	235	99.52	270	2.89

### DNA extraction and PCR amplification

To obtain high total DNA yield, a half filter was cut into small pieces by scissor. Genomic DNA was extracted using PowerSoil DNA isolation kit (MoBio, USA) according to manufacturer's protocols. The internal transcribed spacer 1 (ITS1) region of the fungal ribosomal RNA gene was amplified by PCR (95°C for 3 min, followed by 35 cycles at 95°C for 30 s, 55°C for 40 s, and 72°C for 45 s, and a final extension at 72°C for 10 min) using primers ITS5 (5′-barcode-GGAAGTAAAAGTCGTAACAAGG-3′) and ITS2 (5′-GCTGCGTTCTTCATCGATGC-3′; Bellemain et al., 2010), where barcode is an 8-base sequence unique to each sample. PCR amplification was conducted using high fidelity TransStart Fastpfu DNA Polymerase (Transgen, China). PCR reactions were performed in triplicate in 20 μL mixtures containing 4 μL of 5 × FastPfu Buffer, 2 μL of 2.5 mM dNTPs, 0.8 μL of each primer (5 μM), 0.4 μL of FastPfu Polymerase, and 10 ng of template DNA.

### Illumina MiSeq sequencing

Amplicons were purified using AxyPrep DNA Gel Extraction Kit (Axygen, USA) according to the manufacturer's instructions and quantified using QuantiFluor™-ST (Promega, USA). Purified amplicons were pooled in equimolar and paired-end sequenced (2 × 250 bp) on an Illumina MiSeq platform according to the standard protocols. The raw reads were deposited into the NCBI Sequence Read Archive (SRA) database under accession number SRP068265.

### Processing of sequencing data

Raw files were demultiplexed, quality-filtered using QIIME (version 1.18; Caporaso et al., 2010) with the following criteria: (i) The 300 bp reads were truncated at any site receiving an average quality score <20 over a 50-bp sliding window, discarding the truncated reads that were shorter than 50 bp; (ii) exact barcode matching, two nucleotide mismatch in primer matching, reads containing ambiguous characters were removed; (iii) only sequences that overlap longer than 10 bp were assembled according to their overlap sequence. Reads which could not be assembled were discarded.

### Statistical analyses

All samples were normalized at the same sequence depth (9840 reads). Operational taxonomic units (OTUs) were clustered with 97% similarity cutoff using UPARSE (version 7.1, http://drive5.com/uparse/); chimeric sequences were identified and removed using UCHIME. The OTUs were used as a basis for calculating alpha diversity and beta diversity metrics. The taxonomy of each ITS1 sequence was analyzed by RDP Classifier (Wang et al., 2007) against the Unite database (Release 7.0, http://unite.ut.ee/index.php; Kõljalg et al., 2013) using confidence threshold of 70%. Statistical analyses of the OTU diversity of each air sample via Chao1, Good's coverage estimator, and Shannon's index (*H*′) were performed using QIIME 1.8.0 software (Caporaso et al., 2010). Statistical comparisons of Chao1 and Shannon indices among different samples were made by one-way analysis of variance (ANOVA) using Statistical package for the social sciences (SPSS, version 23.0). An analysis of similarities (ANOSIM) was performed using QIIME 1.8.0 software (Caporaso et al., 2010) to determine whether various types of air samples had significantly different fungal communities. Metastats analysis (White et al., 2009) was used to identify the significantly different fungal genera in different types of air samples using Mothur software (Version 1.35.1). The program Metastats can use count data from annotated sequences to compare two populations in order to detect differentially abundant features and then produce a tab-delimited table displaying the mean relative abundance of a feature, variance, and standard error together with a *p*-value and *q*-value to describe significance of the detected variations (http://metastats.cbcb.umd.edu/).

## Results

### Airborne fungal richness and diversity

A total of 797,040 reads with 9840 reads per sample passed quality filtering, subsampled, and were clustered into 1633 unique OTUs (97% sequence similarity). The alpha diversity in each sample was estimated by Chao1, Good's coverage estimator, and Shannon's indices (Table 1). The rarefaction curves and Shannon index curves indicated that high-throughput sequencing captured the dominant phylotypes (Figure S1). On average, there were 94 (from 34 to 216), 155 (from 59 to 285), and 153 (from 80 to 244) OTUs in samples of PM2.5, PM10, and TSP, respectively.

**Figure 1:**
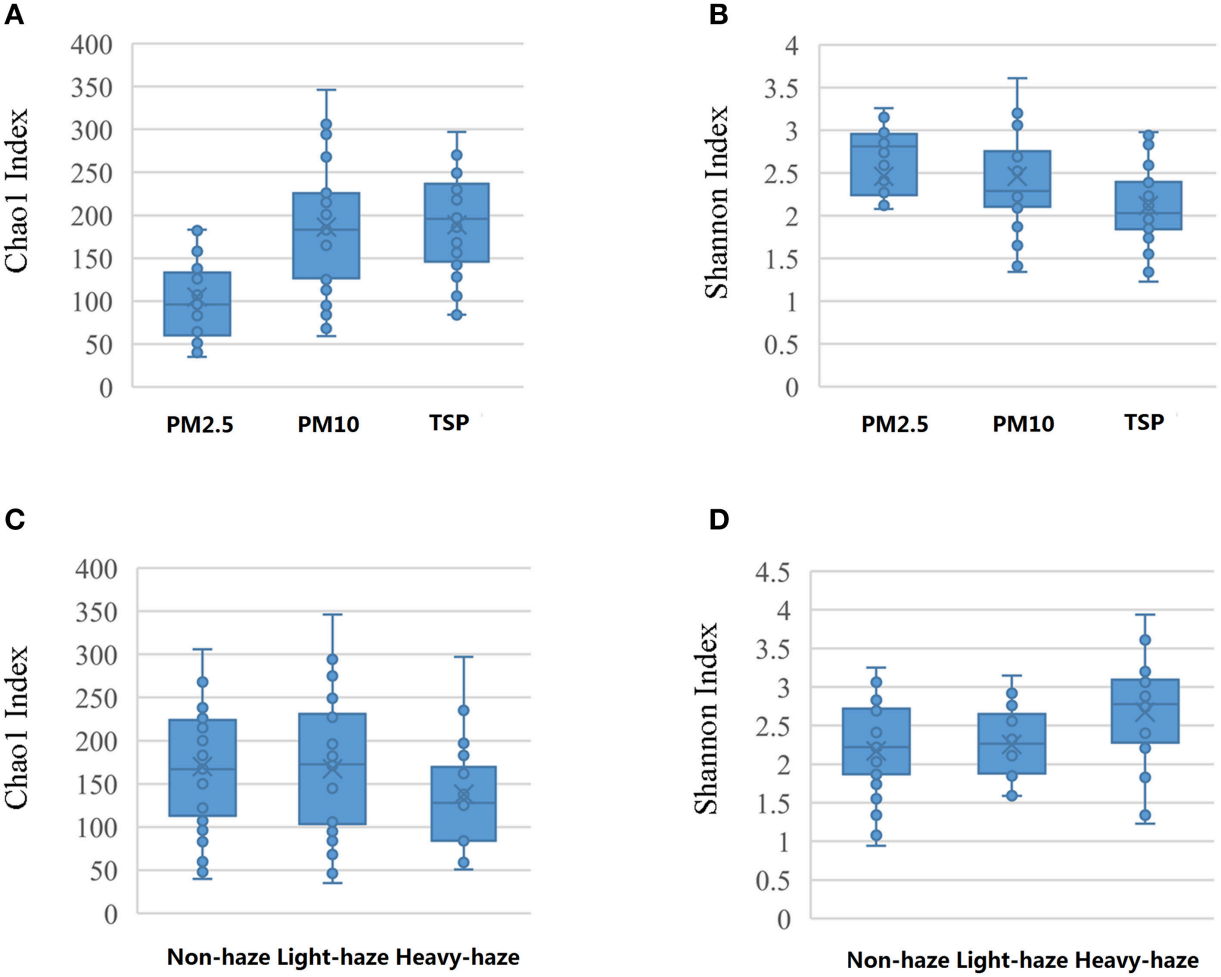
**Statistical comparisons of Chao1 and Shannon indices among three PM types (PM2.5, PM10, and TSP) and three air quality levels (non-haze, light-haze, and heavy-haze). (A)** Values of Chao1 in three particulate matter samples; **(B)** Values of Shannon index in three particulate matter samples; **(C)** Values of Chao1 in three air quality levels; **(D)** Values of Shannon index in three air quality levels.

ANOVA showed that TSP and PM10 samples had higher Chao1-values as compared with PM2.5 samples (*p* < 0.05), which indicated higher richness in TSP and PM10 samples. A relatively lower Shannon index in PM10 and TSP samples was also observed, but there was no significant difference (*p* > 0.05). Samples in heavy-haze days showed a higher value of Shannon index, which indicated a higher diversity than non-haze and light-haze days (*p* < 0.05), whereas there was no significant difference between non-haze and light-haze days (Figure 1).

### Taxonomic composition of airborne fungal community

**Figure 2:**
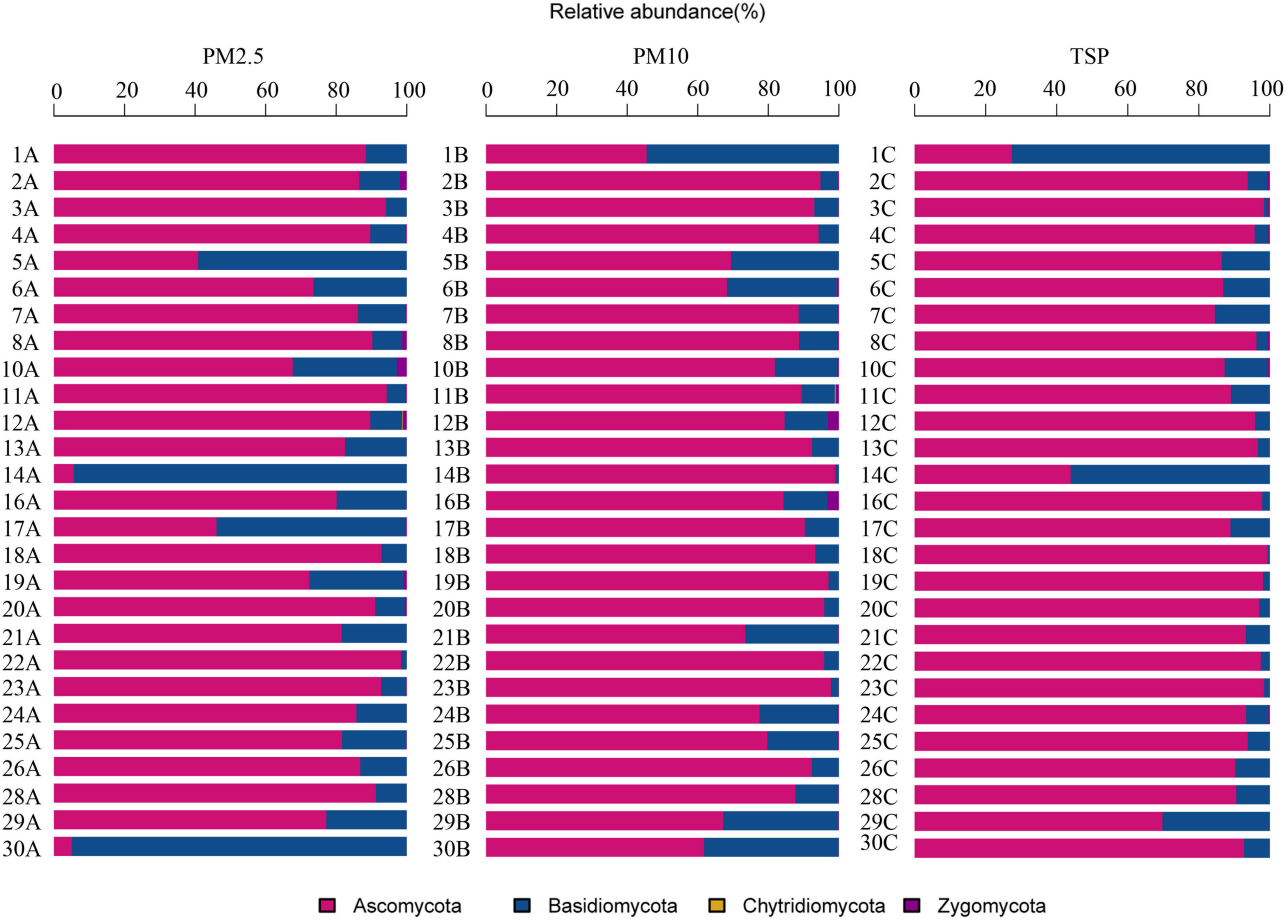
**The relative abundances of different phyla in 81 samples**.

**Figure 3:**
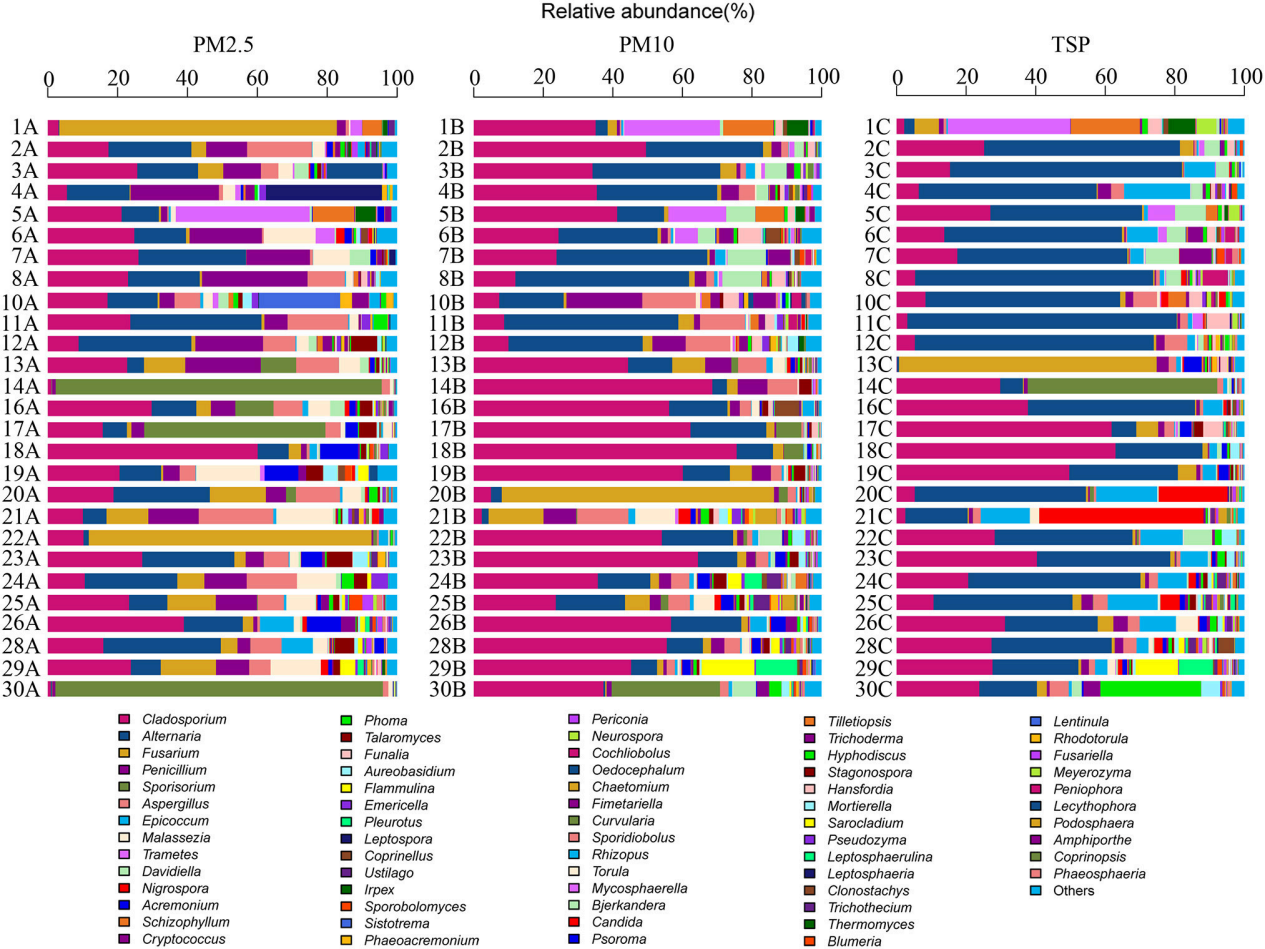
**The relative abundances of different genera in 81 samples**.

A total of four phyla, 71 orders, and 368 genera were identified in the present study. The airborne fungal community was dominated by the Ascomycota, followed by the Basidiomycota, Zygomycota, and Chytridiomycota (Figure 2). The dominant orders were Pleosporales (29.39%), Capnodiales (27.96%), Eurotiales (10.64%), and Hypocreales (9.01%; Figure S2), which belonged to Ascomycota. The abundant orders in Basidiomycota included the Ustilaginales (4.99%), Polyporales (3.57%), and Agaricales (2.78%; Figure S2). Two genera *Cladosporium* and *Alternaria* were the most abundant in fungal community, followed by *Fusarium, Penicillium, Sporisorium*, and *Aspergilus* (Figure 3).

*Cladosporium* and *Alternaria* were two dominant genera in all types of PM samples. Unlike in PM2.5 and PM10 samples, *Alternaria* was more abundant than *Cladosporium* in TSP samples. *Fusarium* was the third abundant genus in PM2.5 and PM10 samples and the fourth abundant genus in TSP samples. *Epicoccum* was one of the dominant genera in TSP samples, whereas there was only a few *Epicoccum* in PM2.5 and PM10 samples. *Sporisorium* showed more abundant in PM2.5 samples. The top three genera in PM2.5 and PM10 samples were same (i.e., *Cladosporium, Alternaria*, and *Fusarium*; Table S2).

In addition, *Cladosporium* and *Alternaria* were two dominant genera in non-haze and light-haze days, whereas, *Alternaria* was most abundant in heavy-haze days. *Sporisorium* showed more abundant in none-haze and light-haze days but not (<1%) in heavy-haze days. *Trametes* was the fourth abundant in non-haze days but not in both light-haze and heavy-haze days. Fifteen dominant genera accounted for 82% of total reads and more diverse genera were observed in heavy-haze days (Table S3).

### Airborne fungal communities associated with different PMs and air quality levels

**Figure 4:**
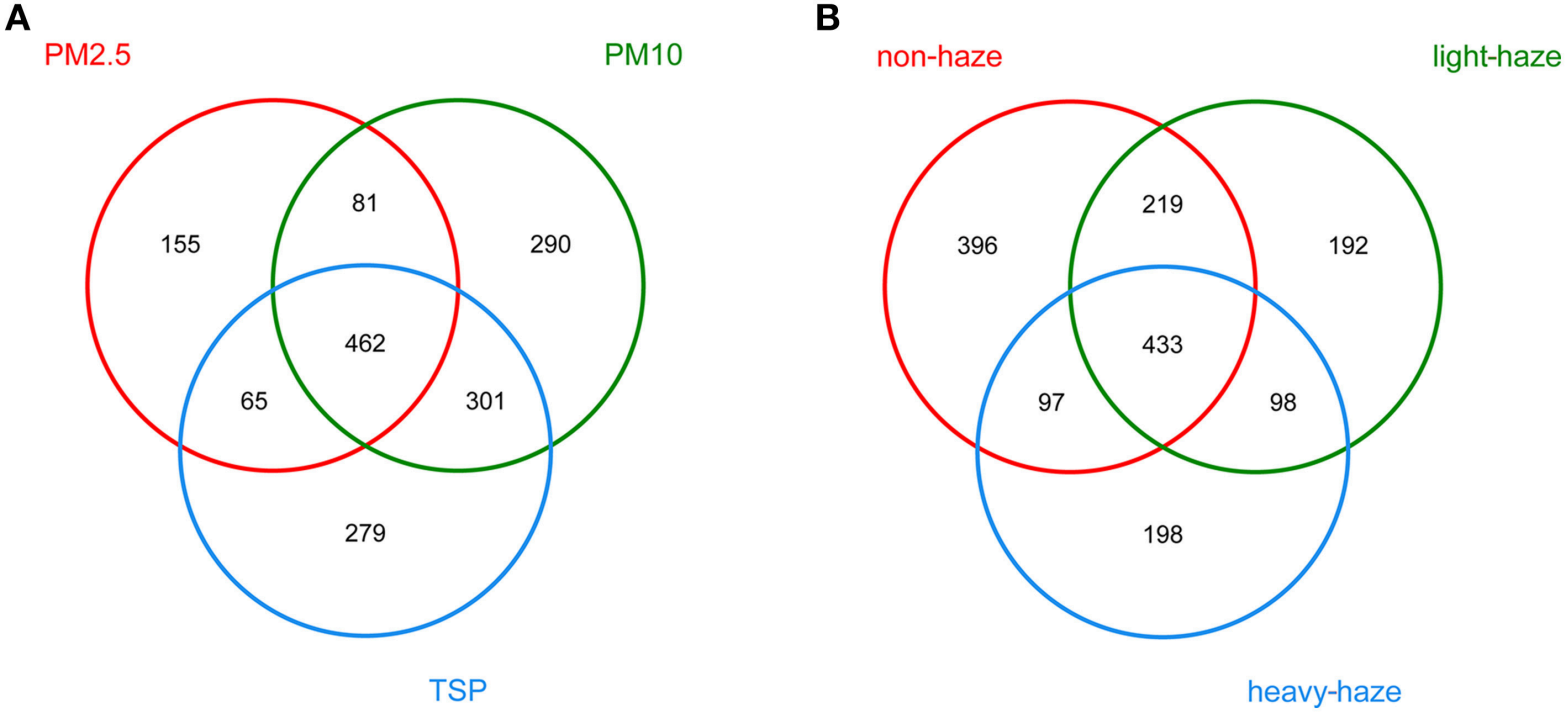
**Venn diagrams illustrating the number of unique and shared OTUs among (A) three PM types (PM2.5, PM10, and TSP) and (B) three air quality levels (non-haze, light-haze, and heavy-haze)**.

ANOSIM (*R* = 0.175, *p* = 0.001) indicated that there was a significant effect of particle size on airborne fungal community composition and a Venn diagram showed that 462 OTUs were shared among PM2.5, PM10, and TSP (Figure 4A). Moreover, there was a significant difference in fungal communities among non-haze, light-haze, and heavy-haze samples (*R* = 0.076, *p* = 0.006). In addition, the Venn diagram showed that there were 433 OTUs shared among non-haze, light-haze, and heavy-haze days (Figure 4B).

**Figure 5:**
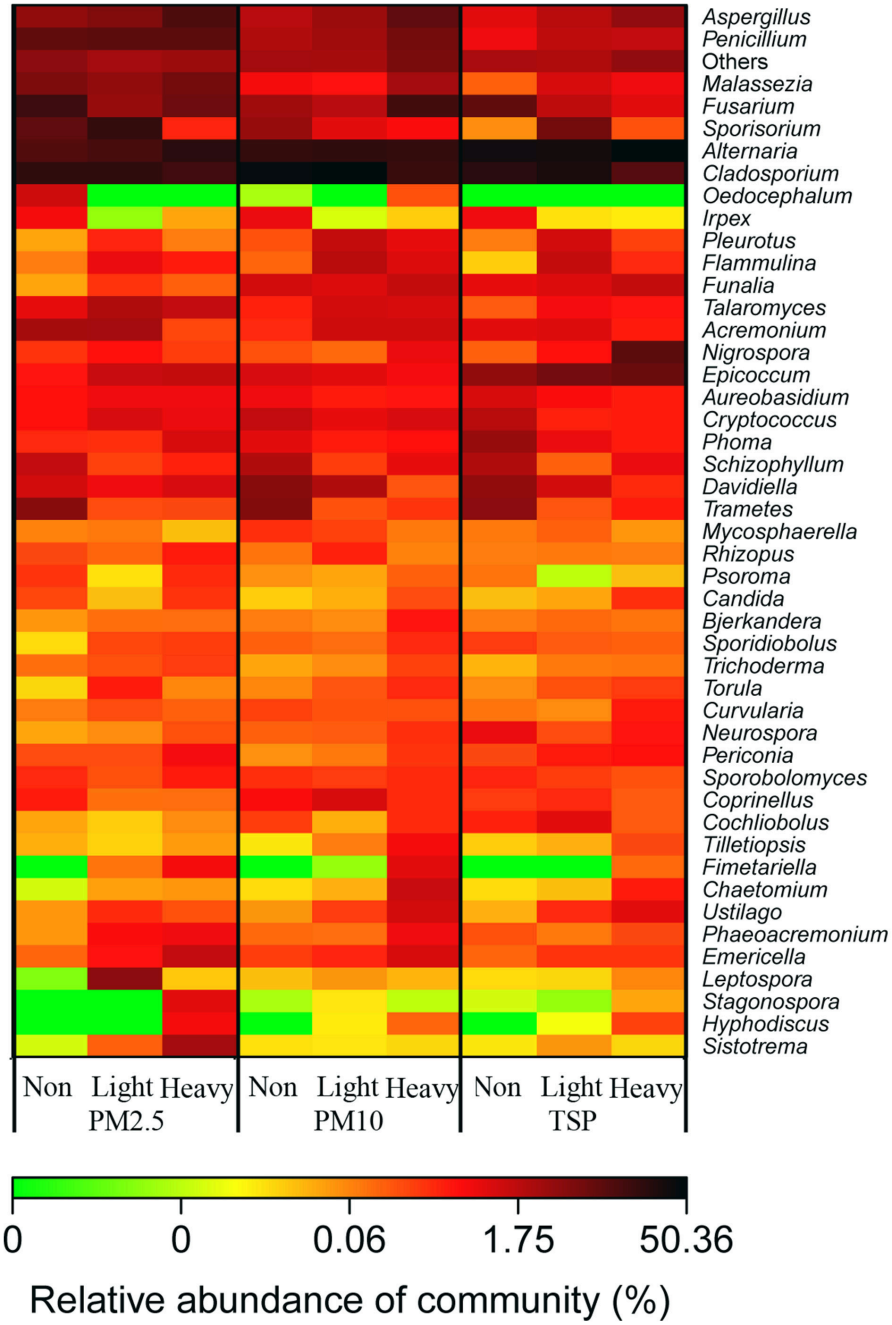
**A heatmap diagram showing the distribution of fungal genera in three PM types (PM2.5, PM10, and TSP) and three air quality levels (non-haze, light-haze, and heavy-haze)**.

A heatmap diagram illustrated the distributions of abundant fungal genera in PMs collected from various air quality days (Figure 5). For examples, *Aspergillus* increased as the haze got heavy and decreased as the particle got bigger; *Penicillium* and *Malassezia* were more abundant in PM2.5 samples and increased during heavy-haze days, especially in PM2.5 and PM10 samples; *Alternaria* was more abundant in TSP samples when compared with PM10 and PM2.5 samples; *Cladosporium* was less during heavy-haze days; *Fusarium* was more abundant in PM2.5 and TSP samples during non-haze days, whereas it is most abundant in PM10 samples during heavy-haze days; *Epicoccum* showed significant abundance in TSP samples.

Metastats analysis demonstrated that some fungal genera showed significant differences among samples of PM2.5, PM10, and TSP. For example, many fungal genera, including *Alternaria, Penicillium, Aspergillus, Epicoccum, Malassezia, Nigrospora, Talaromyces, Funalia, Emericella, Neurospora*, and *Oedocephalum*, showed significant differences between samples of PM2.5 and TSP (Table 2). In addition, some fungal genera showed significant differences among non-haze, light-haze, and heavy-haze days. For example, *Cladosporium, Aspergillus, Trametes, Davidiella, Schizophyllum, Nigrospora, Talaromyces, Flammulina, Emericella, Ustilago, Irpex, Phaeoacremonium, Periconia, Chaetomium, Fimetariella, Torula, Mycosphaerella, Bjerkandera, Tilletiopsis, Trichoderma*, and *Hyphodiscus* showed significant differences between non-haze and heavy-haze days (Table 3).

**Table 2:** **Metastats analysis showing the fungal genera which are significantly different among three PM types**.

**PM2.5 and PM10 samples (*p*-value)**	**PM2.5 and TSP samples (*p*-value)**	**PM10 and TSP samples (*p*-value)**
*Cladosporium* (0.001)	*Alternaria* (0.002)	*Cladosporium* (0.003)
*Penicillium* (0.004)	*Penicillium* (0.001)	*Alternaria* (0.003)
*Malassezia* (0.001)	*Aspergillus* (0.001)	*Penicillium* (0.007)
*Funalia* (0.001)	*Epicoccum* (0.001)	*Aspergillus* (0.016)
*Chaetomium* (0.014)	*Malassezia* (0.001)	*Epicoccum* (0.001)
*Mycosphaerella* (0.005)	*Nigrospora* (0.043)	*Emericella* (0.006)
	*Talaromyces* (0.007)	*Periconia* (0.007)
	*Funalia* (0.002)	*Oedocephalum* (0.001)
	*Emericella* (0.003)	*Mycosphaerella* (0.013)
	*Neurospora* (0.013)	
	*Oedocephalum* (0.001)	

**Table 3:** **Metastats analysis showing the fungal genera which are significantly different among three air quality levels**.

**Non-haze and light-haze (*p*-value)**	**Non-haze and heavy-haze (*p*-value)**	**Light-haze and heavy-haze (*p*-value)**
*Fusarium* (0.04)	*Cladosporium* (0.003)	*Cladosporium* (0.001)
*Trametes* (0.001)	*Aspergillus* (0.001)	*Aspergillus* (0.001)
*Davidiella* (0.03)	*Trametes* (0.006)	*Nigrospora* (0.02)
*Schizophyllum* (0.02)	*Davidiella* (0.001)	*Acremonium* (0.045)
*Talaromyces* (0.03)	*Schizophyllum* (0.03)	*Schizophyllum* (0.036)
*Flammulina* (0.015)	*Nigrospora* (0.005)	*Emericella* (0.006)
*Emericella* (0.006)	*Talaromyces* (0.05)	*Neurospora* (0.047)
*Pleurotus* (0.05)	*Flammulina* (0.001)	*Oedocephalum* (0.001)
*Ustilago* (0.001)	*Emericella* (0.001)	*Chaetomium* (0.002)
*Irpex* (0.006)	*Ustilago* (0.001)	*Fimetariella* (0.03)
*Fimetariella* (0.001)	*Irpex* (0.008)	*Mycosphaerella* (0.02)
*Torula*(0.006)	*Phaeoacremonium* (0.013)	*Bjerkandera* (0.05)
*Psoroma* (0.014)	*Periconia* (0.014)	*Candida* (0.009)
*Hyphodiscus* (0.00001)	*Chaetomium* (0.001)	*Tilletiopsis* (0.025)
	*Fimetariella* (0.001)	*Hyphodiscus* (0.013)
	*Torula* (0.002)	
	*Mycosphaerella* (0.02)	
	*Bjerkandera* (0.03)	
	*Tilletiopsis* (0.003)	
	*Trichoderma* (0.04)	
	*Hyphodiscus* (0.001)	

### The effects of environmental factors on the airborne fungal community

A Pearson correlation analysis was used to examine the relationships between the environmental factors and fungal diversity (Table S4). Values of Shannon index were significantly correlated with RH (*r* = 0.350, *p* < 0.01), PM2.5 (*r* = 0.339, *p* < 0.01), CO (*r* = 0.331, *p* < 0.01), NO_2_ (*r* = 0.264, *p* < 0.05), PM10 (*r* = 0.239, *p* < 0.05), and SO_2_ (*r* = 0.224, *p* < 0.05).

**Figure 6:**
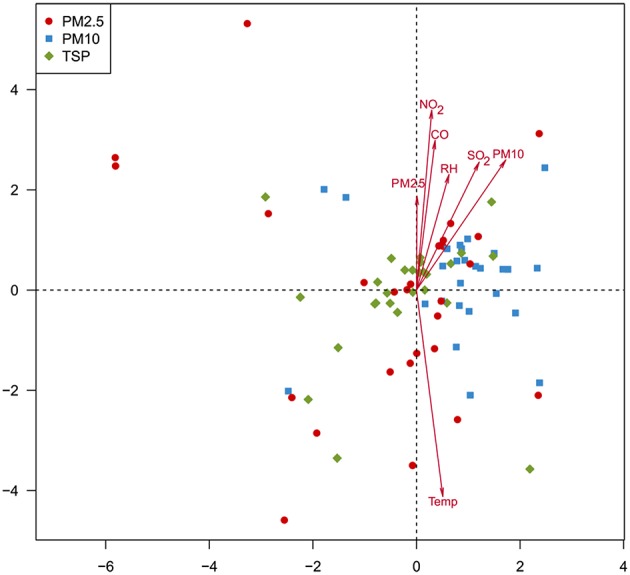
**Canonical correspondence analysis showing the relationships between environmental factors and airborne fungal community composition**.

Canonical correspondence analysis (CCA, Figure 6) and permutation tests (Table S5) were performed to examine the relationships between the environmental factors and fungal community composition. Six environmental factors, including temperature (*r*^2^ = 0.2792, *p* < 0.01), NO_2_ (*r*^2^ = 0.2173, *p* < 0.01), PM10 (*r*^2^ = 0.1692, *p* < 0.01), SO_2_ (*r*^2^ = 0.1382, *p* < 0.01), CO (*r*^2^ = 0.1522, *p* < 0.01), and RH (*r*^2^ = 0.0977, *p* < 0.05), showed significant correlations with airborne fungal community composition.

## Discussion

The aim of the present study was to analyze the airborne fungal community associated with PMs in Beijing using high-throughput sequencing. This study demonstrated the diversity and composition of airborne fungal community from different PM types and air quality levels, which was not clarified before in Beijing.

In the present study, 1633 OTUs (34–285 OTUs per sample) were obtained, which indicated the high richness of airborne fungi associated with PMs. These results are somewhat consistent with a previous study that the fungal OTU numbers associated with the PMs in Rehovot (Israel) were 121–178 OTUs (Dannemiller et al., 2014). Furthermore, the number of OTUs associated with PM10 and TSP was higher than those that were associated with PM2.5 samples. Raisi et al. (2013) found that the highest concentrations of the airborne fungi were determined at aerodynamic diameters between 2.1 and 3.3 μm. In addition, during heavy-haze days, the values of Shannon index were significantly higher than those in non-haze and light-haze days, which are consistent with the concentration of airborne fungi in a culture-dependent study (Li et al., 2015). A high number of particulate matters were observed in haze days as compared with non-haze and light-haze days, which would provide a number of favorable substrates for fungal colonization. Therefore, the fungal diversity in haze days was increased.

The phyla Ascomycota and Basidiomycota dominated the PMs-associated fungal community in Beijing. This result is similar to the previous studies of fungi in the atmosphere (Bowers et al., 2013; Dannemiller et al., 2014; Oh et al., 2014; Womack et al., 2015). Moreover, this result makes sense in light of feature of the Ascomycota, which have single-celled or filamentous vegetative growth forms that are small to become aerosolized, whereas most of the Basidiomycota are too large to be easily aerosolized (Womack et al., 2015). However, some other studies showed that the abundance of Basidiomycota was more than Ascomycota (Barnes et al., 2001; Yamamoto et al., 2014).

In the phylum Ascomycota, Capnodiales and Pleosporales were two abundant orders. Capnodiales have been previously detected in air samples (Oh et al., 2014; Rittenour et al., 2014; Emerson et al., 2015). Pleosporales also have been reported in Kansas City as a broadly cross-reactive and important allergenic fungal order to local residents (Barnes et al., 2001; Sáenz-de-Santamaría et al., 2006; Rittenour et al., 2014). *Alternaria* and *Cladosporium* were the most abundant fungi in our study which is consistent with a previous study (Yamamoto et al., 2012).

Different PMs may affect various regions of the human body: TSP cause skin allergy, PM10 deposit mainly in the head airways, and PM2.5 are more likely to penetrate and deposit deeper in the tracheobronchial and alveolar regions (Brook et al., 2004). Therefore, it is possible to estimate certain fungal infections based on the PM type and haze severity. In the present study, most of the pathogenic and allergenic fungi increased with the air quality levels. For example, the relative abundance of *Aspergillus* significantly increased during heavy-haze days, making human body more susceptible to this pathogenic and allergenic genus in haze days. In addition, some fungal genera which were dominant genera (>1%) and showed significant differences between heavy-haze and non-haze samples may serve as indicators for assessment to the severity of the haze days with other environmental variables. For example, Davidiella and Trametes showed remarkable differences between heavy-haze and non-haze samples, especially in PM10 and TSP samples. The fungal genus *Schizophyllum* also dramatically changed between heavy-haze and non-haze samples in PM2.5 samples. Previous studies also demonstrated that these three genera were prevalent in the atmosphere (Yamamoto et al., 2012, 2014; Oh et al., 2014; Rittenour et al., 2014).

Fungal DNA detected in airborne filter samples may be from spores or fragments of fungal hyphae. The genera *Alternaria* and *Epicoccum* were much abundant in TSP samples as compared with PM2.5 and PM10, which may owe to their large spore diameters (>10 μm; McCartney et al., 1993; Yamamoto et al., 2012). In addition, the airborne fungal composition may be related to the release of fungal spores in the air and the release of spores would depend on environmental factors, such as relative humidity, and wind speed (Pasanen et al., 1991; Kildesø et al., 2003). For example, spores of *Aspergillus* and *Penicillium* were released at air velocity of 0.5 ms^−1^, whereas the release of *Cladosporium* spores required at least a velocity of 1.0 ms^−1^ (Pasanen et al., 1991). Therefore, *Cladosporium* showed less abundant and *Aspergillus* and *Penicillium* were more abundant in heavy-haze days, which is always associated with less wind as compared with non-haze and light-haze days.

Multiple environmental variables may determine the fungal community composition in the atmosphere. PMs were significantly correlated with fungal diversity and community composition in our study, which indicated that PMs might influence the colonization of different fungi. These results are consistent with previous studies that both PM2.5 and PM10 showed positive correlation with culturable fungi concentration (Haas et al., 2013; Liu et al., 2014). In the present study, RH and temperature were also significantly correlated with fungal diversity and community composition. These environmental factors might impact the fungal spores concentration and fungal growth (Troutt and Levetin, 2001; Rodríguez-Rajo et al., 2005; Erkara et al., 2008; Almaguer et al., 2014), and they might show spatial and temporal patterns. For example, the distinct differences of *Alternaria* and *Cladosporium* were observed among the summer and autumn samples, indicating the temporal changes of fungal groups. In addition, some low levels of genera in most of the samples, in our study, showed abundant in a few samples and these irregular changes may owe to the accidental event, such as rainfalls or strong winds (Barnes et al., 2001). Similarly, the airborne fungal communities in Hong Kong were best explained by spatial and temporal factors (Woo et al., 2013). Woo et al. (2013) found that the airborne fungal communities were obviously different among locations (i.e., mid-western urban US locations and Hong Kong locations) and months. In addition, there are many other factors influencing the fungal community in the atmosphere such as wind speed and time of sunlight. Therefore, airborne fungal communities should be surveyed for a long time and their relationships with spatial–temporal factors require further study.

In summary, we have provided an integrated characterization of the airborne fungal community associated with different sizes of particles in Beijing using high-throughput sequencing. Nevertheless, the ecological roles of airborne fungi and their physiological functions remain poorly understood. In the further study, a combination of different technologies, such as traditional culture-based method and metatranscriptomics, may help to answer the pending questions.

## Author contributions

DY, JS, LLZ, HW, and XMF sampled. DY performed the laboratory work and wrote the manuscript. TZ performed the part of laboratory work and revised the manuscript. LYY designed the research and revised the manuscript. DY, YQZ, and HYL analyzed the data for the work. All authors contributed significantly in the preparation of the manuscript. All authors approve of the submission of this manuscript.

### Conflict of interest statement

The authors declare that the research was conducted in the absence of any commercial or financial relationships that could be construed as a potential conflict of interest.

## References

[B1] Adams R. I. Miletto M. Taylor J. W. Bruns T. D. (2013). Dispersal in microbes: fungi in indoor air are dominated by outdoor air and show dispersal limitation at short distances. ISME J. 7, 1262–1273. 10.1038/ismej.2013.28 23426013 PMC3695294

[B2] Adhikari A. Sen M. M. Gupta-Bhattacharya S. Chanda S. (2004). Airborne viable, non-viable, and allergenic fungi in a rural agricultural area of India: a 2-year study at five outdoor sampling stations. Sci. Total Environ. 326, 123–141. 10.1016/j.scitotenv.2003.12.007 15142771

[B3] Almaguer M. Aira M. J. Rodríguez-Rajo F. J. Rojas T. I. (2014). Temporal dynamics of airborne fungi in Havana (Cuba) during dry and rainy seasons: influence of meteorological parameters. Int. J. Biometeorol. 58, 1459–1470. 10.1007/s00484-013-0748-6 24141621

[B4] Awad A. H. A. Gibbs S. G. Tarwater P. M. Green C. F. (2013). Coarse and fine culturable fungal air concentrations in urban and rural homes in Egypt. Int. J. Environ. Res. Public Health. 10, 936–949. 10.3390/ijerph10030936 23466829 PMC3709295

[B5] Barnes C. Tuck J. Simon S. Pacheco F. Hu F. Portnoy J. (2001). Allergenic materials in the house dust of allergy clinic patients. Ann. Allergy Asthma Immunol. 86, 517–523. 10.1016/S1081-1206(10)62899-2 11379802

[B6] Beelen R. Raaschou-Nielsen O. Stafoggia M. Andersen Z. J. Weinmayr G. Hoffmann B.. (2014). Effects of long-term exposure to air pollution on natural-cause mortality: an analysis of 22 European cohorts within the multicentre ESCAPE project. Lancet 383, 785–795. 10.1016/S0140-6736(13)62158-3 24332274

[B7] Bellemain E. Carlsen T. Brochmann C. Coissac E. Taberlet P. Kauserud H. (2010). ITS as an environmental DNA barcode for fungi: an *in silico* approach reveals potential PCR biases. BMC Microbiol. 10:189. 10.1186/1471-2180-10-189 20618939 PMC2909996

[B8] Bowers R. M. Clements N. Emerson J. B. Wiedinmyer C. Hannigan M. P. Fierer N. (2013). Seasonal variability in bacterial and fungal diversity of the near-surface atmosphere. Environ. Sci. Technol. 47, 12097–12106. 10.1021/es402970s 24083487

[B9] Bressi M. Sciare J. Ghersi V. Bonnaire N. Nicolas J. B. Petit J. E. (2013). A one-year comprehensive chemical characterisation of fine aerosol (PM2.5) at urban, suburban and rural background sites in the region of Paris (France). Atmos. Chem. Phys. 13, 7825–7844. 10.5194/acp-13-7825-2013

[B10] Brook R. D. Franklin B. Cascio W. Hong Y. Howard G. Lipsett M.. (2004). Air pollution and cardiovascular disease: a statement for healthcare professionals from the expert panel on population and prevention science of the American Heart Association. Circulation 109, 2655–2671. 10.1161/01.CIR.0000128587.30041.C8 15173049

[B11] Cao C. Jiang W. Wang B. Fang J. Lang J. Tian G.. (2014). Inhalable microorganisms in Beijing's PM2.5 and PM10 pollutants during a severe smog event. Environ. Sci. Technol. 48, 1499–1507. 10.1021/es4048472 24456276 PMC3963435

[B12] Caporaso J. G. Kuczynski J. Stombaugh J. Bittinger K. Bushman F. D. Costello E. K.. (2010). QIIME allows analysis of high-throughput community sequencing data. Nat. Methods 7, 335–336. 10.1038/nmeth.f.303 20383131 PMC3156573

[B13] Cheng Z. Jiang J. Fajardo O. Wang S. Hao J. (2013). Characteristics and health impacts of particulate matter pollution in China (2001–2011). Atmos. Environ. 65, 186–194. 10.1016/j.atmosenv.2012.10.022

[B14] Dannemiller K. C. Lang-Yona N. Yamamoto N. Rudich Y. Peccia J. (2014). Combining real-time PCR and next-generation DNA sequencing to provide quantitative comparisons of fungal aerosol populations. Atmos. Environ. 84, 113–121. 10.1016/j.atmosenv.2013.11.036

[B15] DeFranco E. Hall E. Hossain M. Chen A. Haynes E. N. Jones D.. (2015). Air pollution and stillbirth risk: exposure to airborne particulate matter during pregnancy is associated with fetal death. PLoS ONE 10:e0120594. 10.1371/journal.pone.0120594 25794052 PMC4368103

[B16] Emerson J. B. Keady P. B. Brewer T. E. Clements N. Morgan E. E. Awerbuch J.. (2015). Impacts of flood damage on airborne bacteria and fungi in homes after the 2013 Colorado Front Range flood. Environ. Sci. Technol. 49, 2675–2684. 10.1021/es503845j 25643125

[B17] Erkara I. P. Asan A. Yilmaz V. Pehlivan S. Okten S. S. (2008). Airborne *Alternaria* and *Cladosporium* species and relationship with meteorological conditions in Eskisehir City, Turkey. Environ. Monit. Assess. 144, 31–41. 10.1007/s10661-007-9939-0 17874280

[B18] Evans J. van Donkelaar A. Martin R. V. Burnett R. Rainham D. G. Birkett N. J.. (2013). Estimates of global mortality attributable to particulate air pollution using satellite imagery. Environ. Res. 120, 33–42. 10.1016/j.envres.2012.08.005 22959329

[B19] Fröhlich-Nowoisky J. Pickersgill D. A. Després V. R. Pöschl U. (2009). High diversity of fungi in air particulate matter. Proc. Natl. Acad. Sci. U.S.A. 106, 12814–12819. 10.1073/pnas.0811003106 19617562 PMC2722276

[B20] Gao M. Jia R. Qiu T. Han M. Song Y. Wang X. (2015). Seasonal size distribution of airborne culturable bacteria and fungi and preliminary estimation of their deposition in human lungs during non-haze and haze days. Atmos. Environ. 118, 203–210. 10.1016/j.atmosenv.2015.08.004

[B21] Haas D. Galler H. Luxner J. Zarfel G. Buzina W. Friedl H. (2013). The concentrations of culturable microorganisms in relation to particulate matter in urban air. Atmos. Environ. 65, 215–222. 10.1016/j.atmosenv.2012.10.031

[B22] Huang X. H. Bian Q. Ng W. M. Louie P. K. Yu J. Z. (2014). Characterization of PM2.5 major components and source investigation in suburban Hong Kong: a one year monitoring study. Aerosol. Air Qual. Res. 14, 237–250. 10.4209/aaqr.2013.01.0020

[B23] Hunt A. Abraham J. L. Judson B. Berry C. L. (2003). Toxicologic and epidemiologic clues from the characterization of the 1952 London smog fine particulate matter in archival autopsy lung tissues. Environ. Health Persp. 111, 1209. 10.1289/ehp.6114 12842775 PMC1241576

[B24] Kildesø J. Würtz H. Nielsen K. F. Kruse P. Wilkins K. Thrane U.. (2003). Determination of fungal spore release from wet building materials. Indoor Air 13, 148–155. 10.1034/j.1600-0668.2003.00172.x 12756008

[B25] Kim S. W. Yoon S. C. Jefferson A. Ogren J. A. Dutton E. G. Won J. G. (2005). Aerosol optical, chemical and physical properties at Gosan, Korea during Asian dust and pollution episodes in 2001. Atmos. Environ. 39, 39–50. 10.1016/j.atmosenv.2004.09.056

[B26] Kõljalg U. Nilsson R. H. Abarenkov K. Tedersoo L. Taylor A. F. Bahram M.. (2013). Towards a unified paradigm for sequence-based identification of fungi. Mol. Ecol. 22, 5271–5277. 10.1111/mec.12481 24112409

[B27] Kuhn T. Biswas S. Fine P. M. Geller M. Sioutas C. (2005). Physical and chemical characteristics and volatility of PM in the proximity of a light-duty vehicle freeway. Aerosol. Sci. Technol. 39, 347–357. 10.1080/027868290930024

[B28] Li K. Dong S. Wu Y. Yao M. (2010). Comparison of the biological content of air samples collected at ground level and at higher elevation. Aerobiologia (Bologna) 26, 233–244. 10.1007/s10453-010-9159-x

[B29] Li Y. Fu H. Wang W. Liu J. Meng Q. Wang W. (2015). Characteristics of bacterial and fungal aerosols during the autumn haze days in Xi'an, China. Atmos. Environ. 122, 439–447. 10.1016/j.atmosenv.2015.09.070

[B30] Li Z. Gu X. Wang L. Li D. Xie Y. Li K. (2013). Aerosol physical and chemical properties retrieved from ground-based remote sensing measurements during heavy haze days in Beijing winter. Atmos. Chem. Phys. 13, 10171–10183. 10.5194/acp-13-10171-2013

[B31] Liu Z. Li A. Hu Z. Sun H. (2014). Study on the potential relationships between indoor culturable fungi, particle load and children respiratory health in Xi'an, China. Build. Environ. 80, 105–114. 10.1016/j.buildenv.2014.05.029

[B32] McCartney H. A. Schmechel D. Lacey M. E. (1993). Aerodynamic diameter of conidia of *Alternaria* species. Plant pathol. 42, 280–286. 10.1111/j.1365-3059.1993.tb01501.x

[B33] Oh S. Y. Fong J. J. Park M. S. Chang L. Lim Y. W. (2014). Identifying airborne fungi in Seoul, Korea using metagenomics. J. Microbiol. 52, 465–472. 10.1007/s12275-014-3550-1 24723107

[B34] Pasanen A. L. Pasanen P. Jantunen M. J. Kalliokoski P. (1991). Significance of air humidity and air velocity for fungal spore release into the air. Atmos. Environ. A Gen. Top. 25, 459–462. 10.1016/0960-1686(91)90316-Y

[B35] Pope C. A. III Ezzati M. Dockery D. W. (2009). Fine-particulate air pollution and life expectancy in the United States. N. Engl. J. Med. 360, 376–386. 10.1056/NEJMsa0805646 19164188 PMC3382057

[B36] Putaud J. P. Raes F. Van Dingenen R. Brüggemann E. Facchini M. C. Decesari S. (2004). A European aerosol phenomenology—2: chemical characteristics of particulate matter at kerbside, urban, rural and background sites in Europe. Atmos. Environ. 38, 2579–2595. 10.1016/j.atmosenv.2004.01.041

[B37] Raisi L. Aleksandropoulou V. Lazaridis M. Katsivela E. (2013). Size distribution of viable, cultivable, airborne microbes and their relationship to particulate matter concentrations and meteorological conditions in a Mediterranean site. Aerobiologia (Bologna) 29, 233–248. 10.1007/s10453-012-9276-9

[B38] Rittenour W. R. Ciaccio C. E. Barnes C. S. Kashon M. L. Lemons A. R. Beezhold D. H.. (2014). Internal transcribed spacer rRNA gene sequencing analysis of fungal diversity in Kansas City indoor environments. Environ. Sci. Process. Impact. 16, 33–43. 10.1039/C3EM00441D 24258337 PMC3966654

[B39] Rodríguez-Rajo F. J. Iglesias I. Jato V. (2005). Variation assessment of airborne Alternaria and Cladosporium spores at different bioclimatical conditions. Mycol. Res. 109, 497–507. 10.1017/S0953756204001777 15912938

[B40] Sáenz-de-Santamaría M. Postigo I. Gutierrez-Rodríguez A. Cardona G. Guisantes J. A. Asturias J.. (2006). The major allergen of *Alternaria alternata* (Alt a 1) is expressed in other members of the Pleosporaceae family. Mycoses 49, 91–95. 10.1111/j.1439-0507.2006.01195.x 16466440

[B41] Troutt C. Levetin E. (2001). Correlation of spring spore concentrations and meteorological conditions in Tulsa, Oklahoma. Int. J. Biometeorol. 45, 64–74. 10.1007/s004840100087 11513049

[B42] Wang Q. Garrity G. M. Tiedje J. M. Cole J. R. (2007). Naive Bayesian classifier for rapid assignment of rRNA sequences into the new bacterial taxonomy. Appl. Environ. Microbiol. 73, 5261–5267. 10.1128/AEM.00062-07 17586664 PMC1950982

[B43] Wang Y. Zhuang G. Sun Y. An Z. (2006). The variation of characteristics and formation mechanisms of aerosols in dust, haze, and clear days in Beijing. Atmos. Environ. 40, 6579–6591. 10.1016/j.atmosenv.2006.05.066

[B44] White J. R. Nagarajan N. Pop M. (2009). Statistical methods for detecting differentially abundant features in clinical metagenomic samples. PLoS Comput. Biol. 5:e1000352. 10.1371/journal.pcbi.1000352 19360128 PMC2661018

[B45] Womack A. M. Artaxo P. E. Ishida F. Y. Mueller R. C. Saleska S. R. Wiedemann K. T. (2015). Characterization of active and total fungal communities in the atmosphere over the Amazon rainforest. Biogeosci. Discuss. 12, 7177–7207. 10.5194/bgd-12-7177-2015

[B46] Woo A. C. Brar M. S. Chan Y. Lau M. C. Leung F. C. Scott J. A. (2013). Temporal variation in airborne microbial populations and microbially-derived allergens in a tropical urban landscape. Atmos. Environ. 74, 291–300. 10.1016/j.atmosenv.2013.03.047

[B47] Yamamoto N. Bibby K. Qian J. Hospodsky D. Rismani-Yazdi H. Nazaroff W. W.. (2012). Particle-size distributions and seasonal diversity of allergenic and pathogenic fungi in outdoor air. ISME J. 6, 1801–1811. 10.1038/ismej.2012.30 22476354 PMC3446800

[B48] Yamamoto N. Nazaroff W. W. Peccia J. (2014). Assessing the aerodynamic diameters of taxon-specific fungal bioaerosols by quantitative PCR and next-generation DNA sequencing. J. Aerosol Sci. 78, 1–10. 10.1016/j.jaerosci.2014.08.007

[B49] Yang F. Kawamura K. Chen J. Ho K. Lee S. Gao Y. (2016). Anthropogenic and biogenic organic compounds in summertime fine aerosols (PM2.5) in Beijing, China. Atmos. Environ. 124, 166–175. 10.1016/j.atmosenv.2015.08.095

[B50] Zhang J. Mauzerall D. L. Zhu T. Liang S. Ezzati M. Remais J. V. (2010). Environmental health in China: progress towards clean air and safe water. Lancet 375, 1110–1119. 10.1016/S0140-6736(10)60062-1 20346817 PMC4210128

[B51] Zhang Q. He K. Huo H. (2012). Policy: cleaning China's air. Nature 484, 161–162. 10.1038/484161a 22498609

