# Supplementary material for: Diversity and Composition of Airborne Fungal Community Associated with Particulate Matters in Beijing during Haze and Non-haze Days
Source: Front Microbiol. 2016 Apr 14;7:487. doi: 10.3389/fmicb.2016.00487 (PMC4830834; doi:10.3389/fmicb.2016.00487)
Supplement: Supplementary file 1 [file Table1.DOCX]

**Table S1 | Air conditions of 27 sampling dates investigated in the present study.**

| Date | AQI | PM2.5  (μg/m³) | PM10  (μg/m³) | SO_2_  (μg/m³) | NO_2_  (μg/m³) | CO  (μg/m³) | Temperature  (°C) | Relative Humidity  (%) |
| --- | --- | --- | --- | --- | --- | --- | --- | --- |
| 6/2/2014 | 45 | 31 | 43 | 2 | 26 | 5 | 22 | 53 |
| 6/3/2014 | 63 | 45 | 62 | 13 | 41 | 6 | 26 | 52 |
| 6/4/2014 | 83 | 61 | 90 | 9 | 45 | 8 | 26 | 37 |
| 6/5/2014 | 104 | 78 | 93 | 11 | 41 | 8 | 28 | 43 |
| 6/7/2014 | 32 | 18 | 26 | 2 | 27 | 3 | 24 | 76 |
| 6/11/2014 | 46 | 28 | 41 | 3 | 32 | 7 | 24 | 68 |
| 6/12/2014 | 77 | 56 | 56 | 6 | 39 | 5 | 28 | 43 |
| 6/26/2014 | 109 | 82 | 51 | 4 | 33 | 10 | 27 | 59 |
| 7/3/2014 | 269 | 223 | 128 | 4 | 40 | 13 | 28 | 74 |
| 7/4/2014 | 216 | 165 | 100 | 4 | 38 | 11 | 30 | 66 |
| 7/5/2014 | 210 | 160 | 155 | 8 | 36 | 14 | 30 | 68 |
| 9/16/2014 | 60 | 43 | 81 | 6 | 70 | 7 | 18 | 58 |
| 9/18/2014 | 104 | 78 | 121 | 4 | 55 | 7 | 20 | 62 |
| 9/21/2014 | 116 | 87 | 140 | 19 | 53 | 17 | 22 | 73 |
| 9/27/2014 | 130 | 99 | 98 | 5 | 61 | 10 | 20 | 60 |
| 9/29/2014 | 43 | 20 | 43 | 10 | 37 | 6 | 15 | 45 |
| 9/30/2014 | 74 | 53 | 96 | 8 | 59 | 8 | 14 | 53 |
| 10/8/2014 | 328 | 278 | 348 | 7 | 100 | 26 | 16 | 84 |
| 10/9/2014 | 352 | 301 | 331 | 9 | 103 | 23 | 17 | 85 |
| 10/12/2014 | 15 | 8 | 182 | 3 | 41 | 3 | 13 | 25 |
| 10/17/2014 | 151 | 115 | 176 | 14 | 105 | 16 | 15 | 56 |
| 10/18/2014 | 303 | 253 | 325 | 29 | 132 | 32 | 17 | 68 |
| 10/19/2014 | 211 | 160 | 218 | 10 | 68 | 12 | 16 | 83 |
| 10/20/2014 | 144 | 110 | 107 | 17 | 65 | 13 | 14 | 59 |
| 10/30/2014 | 213 | 162 | 149 | 19 | 104 | 20 | 13 | 69 |
| 10/31/2014 | 175 | 132 | 142 | 6 | 61 | 18 | 13 | 75 |
| 11/1/2014 | 31 | 10 | 30 | 6 | 30 | 3 | 12 | 21 |
