# Supplementary material for: Diversity and Composition of Airborne Fungal Community Associated with Particulate Matters in Beijing during Haze and Non-haze Days
Source: Front Microbiol. 2016 Apr 14;7:487. doi: 10.3389/fmicb.2016.00487 (PMC4830834; doi:10.3389/fmicb.2016.00487)
Supplement: Supplementary file 2 [file Table2.DOCX]

**Table S2 | Information on the dominant fungal genera (>1%) associated with PM2.5, PM10, and TSP.**

| Genus in PM2.5 | Percent | Genus in PM10 | Percent | Genus in TSP | Percent |
| --- | --- | --- | --- | --- | --- |
| *Cladosporium* | 19% | *Cladosporium* | 37% | *Alternaria* | 38% |
| *Alternaria* | 15% | *Alternaria* | 19% | *Cladosporium* | 21% |
| *Fusarium* | 10% | *Fusarium* | 6% | *Epicoccum* | 6% |
| *Sporisorium* | 10% | *Aspergillus* | 4% | *Fusarium* | 4% |
| *Penicillium* | 9% | *Penicillium* | 4% | *Nigrospora* | 3% |
| *Aspergillus* | 7% | *Davidiella* | 2% | *Davidiella* | 2% |
| *Malassezia* | 5% | *Trametes* | 2% | *Sporisorium* | 2% |
| *Acremonium* | 2% | *Sporisorium* | 2% | *Aspergillus* | 2% |
| *Trametes* | 2% | *Cryptococcus* | 1% | *Trametes* | 2% |
| *Talaromyces* | 2% | *Malassezia* | 1% | *Phoma* | 2% |
| *Leptospora* | 1% | *Funalia* | 1% | *Penicillium* | 1% |
| *Epicoccum* | 1% | *Schizophyllum* | 1% | *Schizophyllum* | 1% |
| *Davidiella* | 1% | *Acremonium* | 1% | *Funalia* | 1% |
| *Schizophyllum* | 1% | *Flammulina* | 1% | *Cryptococcus* | 1% |
| *Sistotrema* | 1% | *Epicoccum* | 1% | *Acremonium* | 1% |
| *Cryptococcus* | 1% | *Talaromyces* | 1% | *Aureobasidium* | 1% |
| *Aureobasidium* | 1% | *Pleurotus* | 1% | *Malassezia* | 1% |
| *Emericella* | 1% | *Coprinellus* | 1% | *Flammulina* | 1% |
| *Phoma* | 1% | *Phoma* | 1% | *Neurospora* | 1% |
| *Oedocephalum* | 1% | *Aureobasidium* | 1% | Others | 8% |
| Others | 9% | *Emericella* | 1% |  |  |
|  |  | Others | 8% |  |  |
