# Supplementary material for: Diversity and Composition of Airborne Fungal Community Associated with Particulate Matters in Beijing during Haze and Non-haze Days
Source: Front Microbiol. 2016 Apr 14;7:487. doi: 10.3389/fmicb.2016.00487 (PMC4830834; doi:10.3389/fmicb.2016.00487)
Supplement: Supplementary file 3 [file Table3.DOCX]

**Table S3 | Information on the dominant fungal genera (>1%) in non-haze, light-haze and heavy-haze samples.**

| Non-haze | Percent | Light-haze | Percent | Heavy-haze | Percent |
| --- | --- | --- | --- | --- | --- |
| *Cladosporium* | 29% | *Cladosporium* | 33% | *Alternaria* | 30% |
| *Alternaria* | 21% | *Alternaria* | 22% | *Cladosporium* | 14% |
| *Fusarium* | 10% | *Sporisorium* | 9% | *Aspergillus* | 8% |
| *Trametes* | 4% | *Penicillium* | 5% | *Fusarium* | 7% |
| *Sporisorium* | 4% | *Aspergillus* | 3% | *Penicillium* | 6% |
| *Penicillium* | 4% | *Epicoccum* | 3% | *Nigrospora* | 4% |
| *Davidiella* | 3% | *Fusarium* | 2% | *Malassezia* | 3% |
| *Aspergillus* | 2% | *Malassezia* | 2% | *Epicoccum* | 3% |
| *Malassezia* | 2% | *Acremonium* | 2% | *Talaromyces* | 1% |
| *Schizophyllum* | 2% | *Flammulina* | 1% | *Funalia* | 1% |
| *Epicoccum* | 2% | *Talaromyces* | 1% | *Emericella* | 1% |
| *Phoma* | 2% | *Leptospora* | 1% | *Sistotrema* | 1% |
| *Cryptococcus* | 1% | *Davidiella* | 1% | *Cryptococcus* | 1% |
| *Acremonium* | 1% | *Pleurotus* | 1% | *Ustilago* | 1% |
| *Aureobasidium* | 1% | Others | 14% | *Phoma* | 1% |
| *Irpex* | 1% |  |  | Others | 18% |
| Others | 11% |  |  |  |  |
