# Supplementary material for: Diversity and Composition of Airborne Fungal Community Associated with Particulate Matters in Beijing during Haze and Non-haze Days
Source: Front Microbiol. 2016 Apr 14;7:487. doi: 10.3389/fmicb.2016.00487 (PMC4830834; doi:10.3389/fmicb.2016.00487)
Supplement: Supplementary file 4 [file Table4.DOCX]

**Table S4 | Pearson Correlation of environmental variables and Shannon index.**

|  | PM2.5 | PM10 | SO_2_ | NO_2_ | CO | Temp | RH | Shannon |
| --- | --- | --- | --- | --- | --- | --- | --- | --- |
| PM2.5 | 1 | 0.845** | 0.356** | 0.690** | 0.877** | -0.035 | 0.690** | 0.339** |
| PM10 | 0.845** | 1 | 0.433** | 0.807** | 0.845** | -0.329** | 0.505** | 0.239* |
| SO2 | 0.356** | 0.433** | 1 | 0.666** | 0.643** | -0.272* | 0.135 | 0.224* |
| NO2 | 0.690** | 0.807** | 0.666** | 1 | 0.843** | -0.511** | 0.421** | 0.264* |
| CO | 0.877** | 0.845** | 0.643** | 0.843** | 1 | -0.239* | 0.625** | 0.331** |
| Temp | -0.035 | -0.329** | -0.272* | -0.511** | -0.239* | 1 | 0.056 | -0.052 |
| RH | 0.690** | 0.505** | 0.135 | 0.421** | 0.625** | 0.056 | 1 | 0.350** |
| Shannon | 0.339** | 0.239* | 0.224* | 0.264* | 0.331** | -0.052 | 0.350** | 1 |

** Correlation is significant at the 0.01 level (2-tailed).

* Correlation is significant at the 0.05 level (2-tailed).
