# Supplementary material for: Diversity and Composition of Airborne Fungal Community Associated with Particulate Matters in Beijing during Haze and Non-haze Days
Source: Front Microbiol. 2016 Apr 14;7:487. doi: 10.3389/fmicb.2016.00487 (PMC4830834; doi:10.3389/fmicb.2016.00487)
Supplement: Supplementary file 5 [file Table5.DOCX]

**Table S5 | Permutation test of Canonical correspondence analysis.**

|  | CCA1 | CCA2 | r^2^ | Pr(>r) |
| --- | --- | --- | --- | --- |
| PM2.5 | 0.08221 | 0.99661 | 0.0581 | 0.099 |
| PM10 | 0.55507 | 0.8318 | 0.1692 | 0.002 |
| SO_2_ | 0.45069 | 0.89268 | 0.1382 | 0.006 |
| NO_2_ | 0.15185 | 0.9884 | 0.2173 | 0.001 |
| CO | 0.18524 | 0.98269 | 0.1522 | 0.003 |
| Temp | 0.03883 | -0.99925 | 0.2792 | 0.001 |
| RH | 0.31033 | 0.95063 | 0.0977 | 0.02 |
