# Supplementary figures and images for: Diversity and Composition of Airborne Fungal Community Associated with Particulate Matters in Beijing during Haze and Non-haze Days
Source: Front Microbiol. 2016 Apr 14;7:487. doi: 10.3389/fmicb.2016.00487 (PMC4830834; doi:10.3389/fmicb.2016.00487)

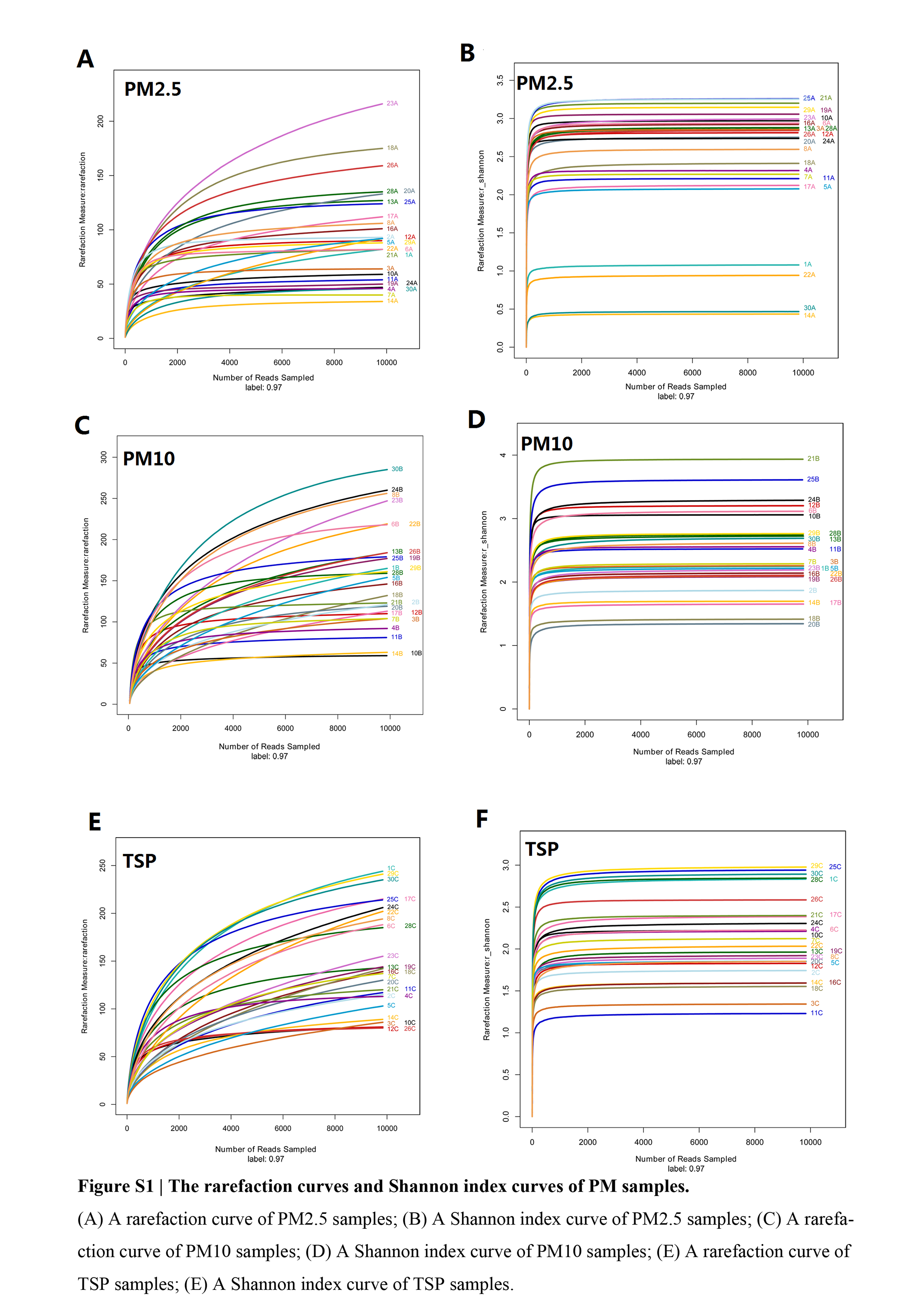

Supplement: Supplementary file 6 [file Image1.TIF]

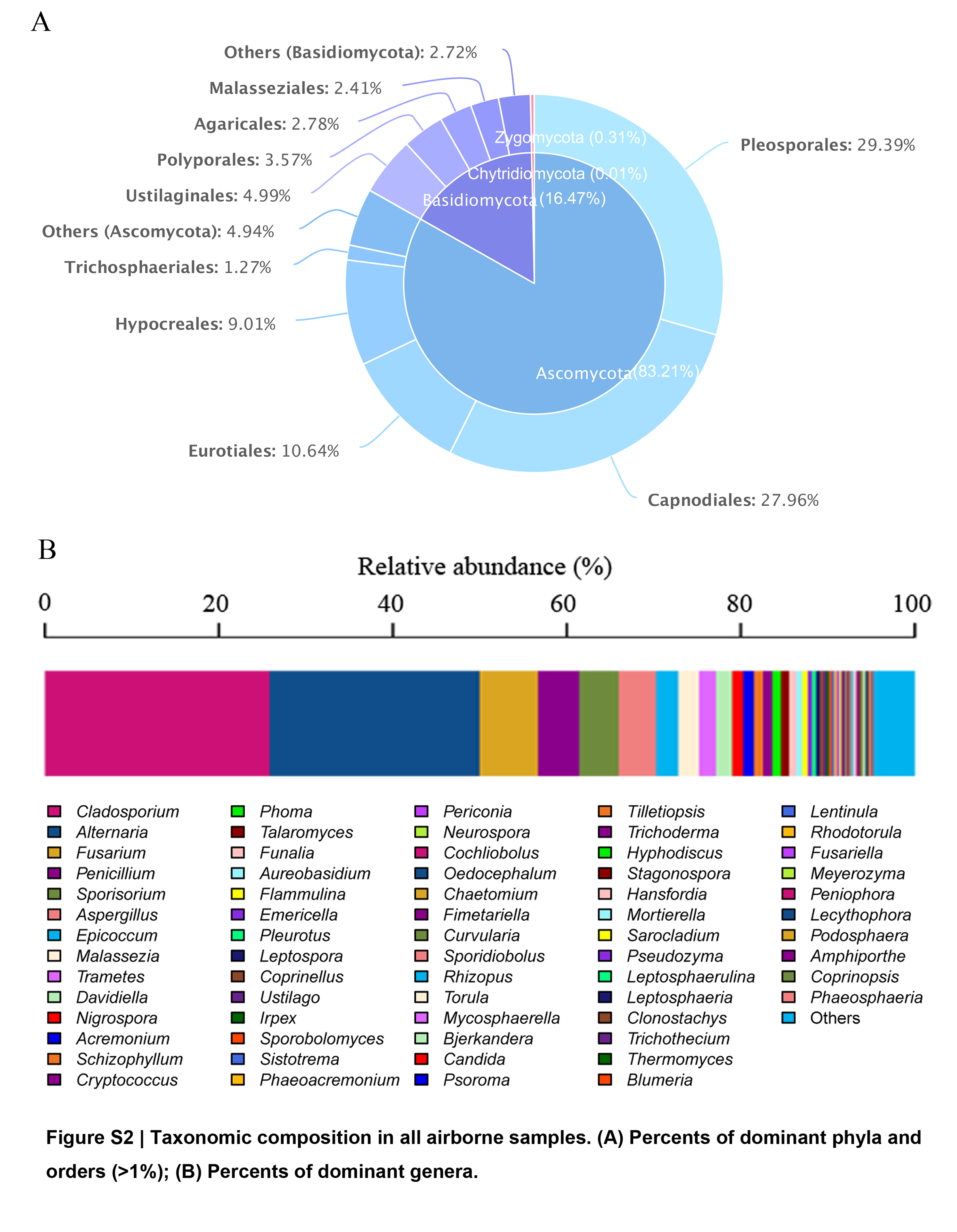

Supplement: Supplementary file 7 [file Image2.tif]
